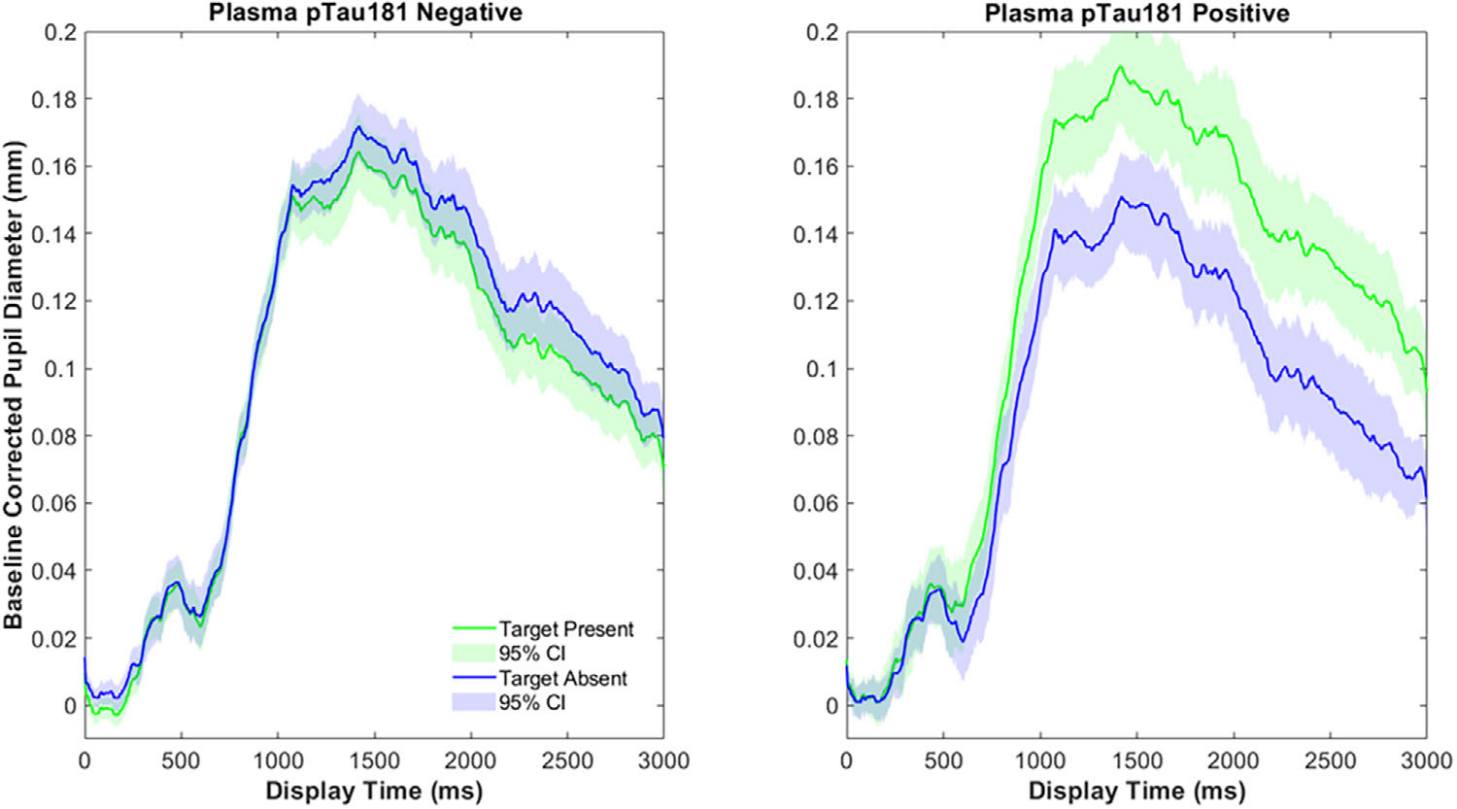# Pupil dilation during visual search is sensitive to plasma pTau181 levels in cognitively healthy older adults

**DOI:** 10.1002/alz.092633

**Published:** 2025-01-03

**Authors:** Elena K Festa, William C Heindel, Camille A. Marangi, Jingjing Zou, Douglas R. Galasko, David P. Salmon, Diane M. Jacobs

**Affiliations:** ^1^ Brown University, Providence, RI USA; ^2^ University of California, San Diego, La Jolla, CA USA

## Abstract

**Background:**

Pathological changes of Alzheimer’s disease (AD) occur in the locus coeruleus (LC) years before clinical symptoms. This may affect LC activity that can be indexed by changes in pupil dilation. AD also disrupts connections between related but functionally distinct cortical areas leading to visual feature binding deficits. Thus, feature binding in individuals with preclinical AD may require additional cognitive effort that engages LC activity. Pupil dilation during conjunctive visual search may therefore be a particularly sensitive marker of preclinical AD.

**Method:**

Sixty‐three cognitively unimpaired older adults from the UCSD Alzheimer’s Disease Research Center (51% men; overall mean age = 78.4 years, education = 17.5 years, and MoCA score = 25.9), performed a conjunctive visual search task while their pupil diameter was continuously recorded. There were 108 trials in which the participant searched for a target stimulus (a black dot moving up‐and‐down) among distractor stimuli that shared target features (white dots moving up‐and‐down and black dots moving left‐and‐right). Search displays contained 1, 3, or 5 stimuli with a target present on half of the trials. Reaction time and accuracy in detecting presence of a target were recorded. Plasma levels of the AD biomarker pTau181 were determined by the National Centralized Repository for Alzheimer’s Disease using Quanterix (v2). Plasma pTau181>4.09 pg/mL was considered AD positive.

**Result:**

AD positive (n = 19) and AD negative (n = 44) groups did not differ on age, education, sex, or MoCA scores. Despite similar behavioral performance in visual search, the groups displayed striking differences in pupillary responses across conditions. Both groups showed more pupil dilation with increasing display size, consistent with the application of cognitive effort. However, only the AD‐positive group showed significantly greater pupil dilation in the target‐present than target‐absent condition. This selective pupil modulation in the AD‐positive group may reflect greater demands associated with maintaining the search template in working memory or with performing template‐matching processes to guide attention.

**Conclusion:**

Results suggest early functional changes in the LC noradrenergic system are detectable in biomarker‐positive cognitively healthy older adults. Pupillary responsivity under task‐induced conjunction search conditions may serve as a particularly sensitive marker of preclinical AD.